# Millet in Bioregenerative Life Support Systems: Hypergravity Resilience and Predictive Yield Models

**DOI:** 10.3390/life15081261

**Published:** 2025-08-07

**Authors:** Tatiana S. Aniskina, Arkady N. Kudritsky, Olga A. Shchuklina, Nikita E. Andreev, Ekaterina N. Baranova

**Affiliations:** 1 N.V. Tsitsin Main Botanical Garden of Russian Academy of Sciences, 127276 Moscow, Russia; oashuklina@gmail.com; 2 Federal State Budgetary Educational Institution of Higher Education “D.F.”, Ustinov Baltic State Technical University “VOENMEH”, 190005 Saint Petersburg, Russia; kudritskiy.arkadiy@mail.ru; 3 Moscow Power Engineering Institute, National Research University, 111250 Moscow, Russia; andreevny@mpei.ru

**Keywords:** *Panicum miliaceum* L., centrifugation, germination, hypergravity, seedling development, yield modeling, bioregenerative life support systems, yield components

## Abstract

The prospects for long-distance space flights are becoming increasingly realistic, and one of the key factors for their implementation is the creation of sustainable systems for producing food on site. Therefore, the aim of our work is to assess the prospects for using millet in biological life support systems and to create predictive models of yield components for automating plant cultivation control. The study found that stress from hypergravity (800 g, 1200 g, 2000 g, and 3000 g) in the early stages of millet germination does not affect seedlings or yield. In a closed system, millet yield reached 0.31 kg/m^2^, the weight of 1000 seeds was 8.61 g, and the yield index was 0.06. The paper describes 40 quantitative traits, including six leaf and trichome traits and nine grain traits from the lower, middle and upper parts of the inflorescence. The compiled predictive regression equations allow predicting the accumulation of biomass in seedlings on the 10th and 20th days of cultivation, as well as the weight of 1000 seeds, the number of productive inflorescences, the total above-ground mass, and the number and weight of grains per plant. These equations open up opportunities for the development of computer vision and high-speed plant phenotyping programs that will allow automatic correction of the plant cultivation process and modeling of the required yield. Predicting biomass yield will also be useful in assessing the load on the waste-free processing system for plant waste at planetary stations.

## 1. Introduction

Interest in space flights to the Moon and Mars continues to grow thanks to initiatives such as the Russian Space Programme [1], NASA’s Artemis program [2], SpaceX’s ambitions to colonize Mars [3], and international cooperation on the Lunar Gateway [4]. However, the logistical and financial challenges associated with maintaining a human presence in space remain a serious obstacle. For example, transporting 1 kg of cargo to the International Space Station costs approximately 6500 USD [5], and a crew of three astronauts consumes about 3 kg of dry food per day [6], which, when extrapolated to long-term space missions, leads to exorbitant costs for interested states and private companies [7]. Therefore, there is a need for sustainable food production on a permanent basis at space stations and planetary bases.

An elucidation of candidate plants for bioregenerative life support systems is actively continuing today [8,9,10,11,12]. The most important crops for maintaining the crew in life support conditions during long-term flights are cereals and grain crops with a high content of available carbohydrates and proteins, which form the basis of nutrition [9,10,11]. However, a list of plants that provide complex nutrition necessary to maintain human health during long-term flights has not yet been compiled. This gap in nutrition is a strong argument for searching for alternative highly productive crops for closed systems.

Proso millet (*Panicum miliaceum* L.) is a drought-resistant crop with C4 photosynthesis, which gives it a low transpiration rate [13,14]. Millet has high nutritional value: it is easily digestible, gluten-free, rich in protein (10–14 g/100 g), carbohydrates, dietary fiber, as well as micronutrients (Fe, Zn, Mg, Mn, K, P) and vitamins (niacin, B group) [14,15]. Compared to other cereals, millet also has a more balanced composition of essential amino acids [16,17]. This crop plays a key role in ensuring food security, especially in regions with poor soils, arid climates, and a lack of irrigation, such as India and African countries [13,18]. Its short growing season (60–100 days) makes millet a promising crop for extreme conditions, including long-term space missions, where it can serve as a backup food source. In addition, its drought resistance allows it to be used in crisis situations involving water shortages [17].

The aim of our study is to develop predictive equations for millet yield components for cultivation in bioregenerative life support systems. The objectives of the experiment are to test the hypothesis that hypergravity does not affect plants in the 10- and 20-day seedling stages or when the grain is fully ripe; to model plant biomass accumulation for future process automation; and to model the dependence of valuable millet traits on linear traits.

## 2. Materials and Methods

### 2.1. Preparation of Plants for the Experiment

From the initial batch, a sample of millet seeds was randomly selected, visually inspected to remove damaged and deformed specimens, and then size-calibrated to minimize the impact of weight variability on germination. To prepare for centrifugation, millet seeds (*Panicum miliaceum* L.) were treated with a fungicide (25 g/L fludioxonil) (Syngenta Italy, Milan, Italy) and washed with distilled water. The seeds were then placed in 10 mL centrifuge tubes filled with water. Each test group was exposed in the centrifuge MPW-310 (MPW Med. instruments, Warsaw, Poland) for 3 h, as this duration ensures complete water saturation of the seeds and coincides with the onset of the first critical metabolic phases. The variants of the experiment were exposure to hypergravity of 800 g (corresponding to 3000 revolutions per minute (rpm) on the angular impeller), 1200 g, 2000 g, 3000 g, control (1 g, without placement in a centrifuge, but also with soaking in water). The selection of these hypergravity levels was determined by the centrifuge’s technical capabilities, where the specified values correspond to standard operating modes (3000, 4000, 5000, and 6000 rpm, respectively), as well as the need to cover a wide range of potential threshold effects. Next, the seeds were sown in 0.5 L technical pots (substrate: peat + perlite with slow-release NPK 15: 9: 12 fertilizer at a rate of 2 g/L). Three plants were sown in each 10 × 10 cm container. Each variant was represented in three replicates of 50 seeds formed by the method of blind selection.

The plants were kept in a phytotron from sowing to harvest. The variants were randomly arranged. Lighting was provided by a system of LEDs (Gauss Elementary 50 W 4495 lm 4000 K) with an intensity of 50 W/m^2^ (LLC Gauss Lighting, Moscow, Russia), 24 h lighting. At the beginning of the growing season, the distance between the lamps and the plants was 1.5 m. By the end of the season, the tips of the millet panicles remained no closer than 15 cm to the light source. The temperature was regulated by an air conditioning system and maintained at 24–28 °C. The relative humidity was within the range of 30–50%. The plants were watered manually as the substrate dried out.

### 2.2. Data Collection

The germination of the samples was assessed. At 10 and 20 days after sowing, 10 plants were cut from each research variant in triplicate. The mass and length of the seedlings were measured. LEKI Electronic Balance B2104 analytical scales (*p* = 0.0001 g) (LEKI Instruments, Helsinki, Finland) and a ruler were used.

**Figure 1:**
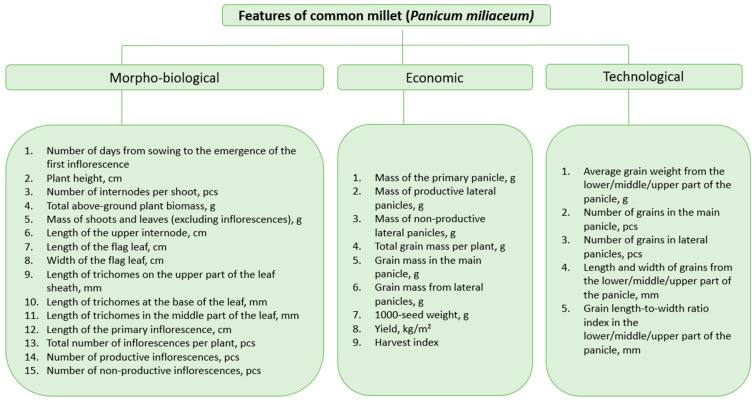
The figure lists all the described characteristics of *Panicum millaceum*.

At the stage of full grain ripeness (according to the Zadoks scale [19]), the morpho-biological, economic, and technological characteristics of the plants were determined (Figure 1). To determine the length of the trichomes and the size of the grain, the leaf and grain were scanned on an Epson Perfection V500 Photo at 600 dpi, and then measurements were taken using ImageJ software (Java 1.8.0) [20].

### 2.3. Statistical Analysis

Statistical analysis was performed using SPSS Statistics 25. The normality of data distribution was tested using the Kolmogorov–Smirnov test. The comparison of means was performed using ANOVA with Duncan’s post hoc test (*p* = 0.05) for parametric criteria and Kruskal–Wallis one-way analysis of variance for non-parametric criteria. Pearson’s correlation analysis with a two-tailed significance test (*p* = 0.01) was used to test relationships between characteristics. For traits with strong relationships, linear regression predictor equations were constructed, indicating quality by coefficients of determination and standard error of estimation. Trait dimensionality reduction was performed using the principal component method with Varimax rotation and Kaiser normalization.

## 3. Results

### 3.1. The Effect of Hypergravity on Plants

The 3 h hypergravity treatment showed no significant effect (*p* > 0.05) on millet germination when comparing experimental variants with the control group, with an average germination rate of 79%.

The mass and length of shoots of plants whose seeds were exposed to hypergravity in a moist state on the 10th and 20th days of the experiment did not differ from the control group. The average shoot mass on day 10 was 0.06 ± 0.02 g at a height of 6.27 ± 1.46 cm; after another 10 days of cultivation, these parameters increased on average by four times (Appendix A, Table A1, Table A2 and Table A3).

The effect of hypergravity in the early stages of seed swelling did not lead to significant differences between the experimental variants in the phase of full ripeness of new seeds [13] (Appendix A). It was found that under phytotron conditions, millet reached an average height of 121.6 ± 21.0 cm and gained a biomass of 6.22 ± 2.63 g, both of which showed a fairly high variation of 41–42%. There was an average of 2.6 ± 1.5 productive inflorescences (coefficient of variation 58%) and 0.2 ± 0.17 non-productive inflorescences. The average grain weight per plant was 0.34 ± 0.29 g, and the yield was 0.3 ± 0.2 kg/m^2^. The weight of 1000 seeds has a low coefficient of variation (8%) and is 8.61 ± 0.65 g.

### 3.2. Predictive Models of Millet Biomass Accumulation on the 10th and 20th Days After Sowing

Selection of models based on the coefficient of determination and standard error of estimation showed that among the 11 models (linear, logarithmic, inverse, quadratic, cubic, composite, power, sigmoid, growth, exponential, logistic) proposed in SPSS Statistics, the linear and quadratic models were the most suitable. In the case of 10- and 20-day seedlings, the coefficient of determination of quadratic equations is closer to 1 and the standard error of estimation of the results obtained is lower (Table 1), i.e., they can more accurately predict biomass accumulation based on such a linear feature as plant height.

The accuracy of the forecasts is quite high for all equations, as the coefficient of determination is greater than 0.7. Regression equations for 10-day plants can explain the variation of 75–76% of the sample elements, and for 20-day plants, it is 81–83% of the sample.

### 3.3. Calculation of Prediction Models for Millet Traits Related to Yield When Grown in Closed Systems

To compile the equations, only traits with strong and reliable (*p* = 0.01) correlations were selected (Table 2).

The weight of 1000 seeds is an important component of yield. The first equation allows predicting up to 65% of the sample volume based on two linear traits: the length of the main inflorescence and the number of all inflorescences on the plant. This is useful for predicting the weight of 1000 seeds long before the seed ripening stage. Adding the trait ‘grain weight in the main inflorescence’ increases the accuracy of the equation and allows describing up to 79.9% of the variation in the predicted value.

The grain weight in the main inflorescence has a high correlation with the number of grains in all inflorescences of the plant. Therefore, the number of grains on the entire plant can be predicted based on the grain weight in the main inflorescence (R^2^ = 0.996, i.e., the relationship is close to functional), and vice versa, the number of grains in the main inflorescence can be predicted based on the total grain weight of the entire plant (the equation describes 99.2% of the variation in the sample).

The principal component method with Varimax rotation and Kaiser normalization showed that the first component explains 24.4% of the variance, the second component explains 16.2%, and the third component explains 12.0%. The three components explain 52.6% of the sample. Component 4 explains 8.9% (with it explaining 61.5), component 5 explains 7.0% (68.5%). Component 1 is the mass of the plant without grain (g), the total number of shoots per plant (pcs), the number of all inflorescences on the shoots, the number of productive inflorescences, the total above-ground mass of the plant, the mass of shoots and leaves without inflorescences, the mass of productive lateral shoots, and the mass and number of grains in lateral shoots. Component 2—mass of the first-order inflorescence, mass of grains from the plant, mass of grains in the first-order inflorescence, mass of 1000 seeds. Component 3 is related to grain parameters—average grain weight from the lower and upper parts of the inflorescence, average grain length from the lower, middle and upper parts of the inflorescence, and average grain width in the upper part of the inflorescence. Component 4 characterizes linear parameters—plant height, length of the upper internode, length of the first-order inflorescence, length of the trichomes of the upper part of the leaf sheath. Component 5 combines the parameters of the flag leaf—length and width.

## 4. Discussion

Millet is highly resistant to a range of adverse factors [16,17,18]. In this study, we found that millet plants aged 10 and 20 days after sowing and, in the phase of full grain ripeness, are resistant to stress due to hypergravity, which affected the initial stages of grain metabolism. There were also no differences in germination compared to the control group. Thus, under natural Earth gravity conditions (1 g) and at a temperature of 20–25 °C, during the first 2–4 h of seed soaking, water enters passively through the micropyle along the water potential gradient (matrix and osmotic potentials). The critical moisture content of millet seeds is 25% [21]. At this time, starch hydration occurs, and the processes of primary metabolism are initiated, and respiratory enzymes are activated. Since the samples in the test tubes with water were in the centrifuge for 3 h, the centrifugal force at 3000, 4000, 5000, and 6000 revolutions per minute accelerated the process of grain saturation with water and the course of biochemical reactions, but this effect was not sufficient to cause significant changes in metabolic processes. In experiments with corn (*Zea mays*), 7-day-old seedlings after 2, 4, and 6 h of treatment responded to hypergravity, and their shoot length was shorter than that of the control samples [22]. When wheat seeds were soaked for 24 h and centrifuged for 10 min with hypergravity ranging from 500 to 2500 g, seedling growth was suppressed with increasing g [23]. An experiment with *Arabidopsis thaliana* over 21 days showed that the sensitivity of plants to the magnitude of g was much less than to the direction of its vector and had little effect on morphological endpoints [24]. Pea seedlings (*Pisum sativum*) after 5 days of growth under constant exposure to hypergravity from 1 g to 1054 g showed no differences in germination, and peas were able to germinate successfully even at 10,000 g [25]. Based on this, it can be assumed that the response to hypergravity is largely determined by the specific characteristics of the plant species.

We encountered a lack of information on the correlation between the applied hypergravity force at different stages of millet germination and subsequent changes in physiological, biochemical, and morphological parameters at all stages of ontogenesis. For example, after 10–12 h of seed soaking, millet reaches a moisture content of 45–55%, and the processes of protein synthesis and transcription of new mRNAs are initiated [26]. After approximately 18–20 h of soaking, the moisture content of the grains increases to 52–54%, and full respiration is activated [27]. At 70% grain moisture content, the embryo grows by stretching (22–24 h of soaking).

The search for strong correlations has led to the conclusion that regression equations can be constructed based on such characteristics as shoot weight, shoot length, weight of 1000 seeds, grain weight, inflorescence length and number, number of productive inflorescences, and number of grains. Accordingly, changes in these parameters will entail changes in the associated traits. The yield components of *Panicum miliaceum* respond directly to adjustments in nutrient supply, water management, and planting density [28]. Studies show that applying nitrogen fertilizers at a dose of 150 kg/ha increases shoot length (up to 94.7 cm) and increases grain yield by approximately 20.6% compared to lower doses [28]. Foliar feeding with humic acid increases plant height, inflorescence length, and yield by approximately 30% compared to the control, with 1000 seeds weighing 5.80 g and approximately 900 grains per inflorescence [29]. Silicon applied under drought and salinity conditions improves seed formation, and maintaining moisture along both the main and lateral roots increases shoot mass [30]. In arid and Mediterranean conditions, shorter irrigation intervals (e.g., 6-day cycles) provide higher yields, and higher plant density (222 plants/m^2^) or an optimal sowing rate of approximately 10 kg/ha are associated with improved shoot size, shoot number, and grain number [28]. The occurrence of drought at the inflorescence stage significantly affects grain yield, reducing the number of panicles and seed weight [14,31], while the grain filling stage is less susceptible to drought [32]. Research by Calamai et al. [33] of 80 millet samples over two years showed a wide range of variability in economically valuable traits. The researchers found that plant height varied from 25 to 111 cm, grain yield from 842 to 3125 kg/ha, total dry biomass from 2.77 to 10.63 kg/ha, yield index 0.25–0.35, and number of days to maturity from 80 to 111 days [32]. Our results are consistent with these varied ranges, but our yield index was 0.06, indicating that millet yielded less than it potentially could have. The reduced yield index compared to the literature data can be attributed to fundamentally different growing conditions—published values are based on open-field cultivation, while our study was conducted in a phytotron. The lower index also results from: limited substrate volume (0.5 L vs. open-field conditions in cited studies), reduced light intensity (compared to Mediterranean climate conditions [32]), and the cultivar’s lack of adaptation to closed systems. In subsequent experiments, these factors will be mitigated through irrigation automation and planting density optimization. Thus, the low yield index primarily reflects suboptimal system parameters rather than crop characteristics.

Our predictive models utilize simple linear and quadratic equations, selected for their high accuracy and low error rates in calculations. These models align well with established biological principles of plant development documented in prior research. The observed correlation between longer millet panicles and increased grain weight reflects fundamental genetic and ontogenetic patterns that enhance yield potential [34]. This relationship is supported by recent studies [35,36], which identified significant positive correlations and shared quantitative trait loci (QTLs) for panicle length and grain mass. This association is modulated by environmental factors and resource allocation patterns—particularly water availability, planting density, and photoperiod, which collectively influence both panicle development and grain filling [37].

The advantage of this work is that the experimental plants were grown to harvest, whereas often studies of the effects of hypergravity end at the seedling stage [38]. We see further prospects in conducting experiments on the effect of hypergravity on different stages of grain swelling, for example, after 6–8 h of soaking—at the moment of activation of the embryo, enzymes, and hormones, and after 12–24 h, when protein synthesis and enzyme activity are at their highest. It is also necessary to assess the influence of high micro- and hypergravity values and closed plant cultivation systems on the phenological dates of millet in order to compile a calendar of continuous crop yields.

As a study limitation, it should be noted that the selected hypergravity levels were determined by equipment specifications. Future research should include a more systematic analysis with evenly distributed gravity values and varied exposure durations. These models are applicable under specific conditions: 50 W/m^2^ continuous lighting, 24–28 °C temperature, and 30–50% relative humidity for millet cultivation. They also require recalibration for different millet cultivars and architectural hybrids.

The selection of morphological parameters (shoot length, number of inflorescences) was based on their suitability for remote monitoring in microgravity conditions using sensor systems and photo/video recording methods. The statistically confirmed correlation between these indicators and yield (R^2^ > 0.7) ensures the reliability of predictive models for operational analysis. Although current models do not account for environmental parameters, their high internal consistency makes them suitable as a methodological foundation for automated plant cultivation management systems, computer vision algorithm development, and high-precision phenotyping. The established relationship between shoot length and biomass (Table 1) enables non-invasive assessment of growth processes and timely adjustment of lighting and irrigation regimes. Predictive models for 1000-seed weight and number of productive inflorescences (Table 2) can be integrated into early yield forecasting systems, which is particularly important for resource planning in space-constrained environments.

Research prospects include real-time integration of environmental parameters (light intensity, temperature), development of adaptive control algorithms, and creation of comprehensive predictive analytics systems. Particularly crucial is biomass dynamics monitoring for calculating loads on recycling systems, maintaining ecological balance, and optimizing resource consumption in closed-loop ecosystems of planetary stations. For closed life-support systems, we additionally propose implementing genetic optimization of cultivars, specifically focusing on breeding compact architecture varieties based on the identified shoot length/yield correlations.

## 5. Conclusions

The studies conducted confirmed the potential of using millet in bioregenerative life support systems, demonstrating its resistance to hypergravity and sufficient yield in closed conditions. The developed predictive models could serve as the foundation for automated millet cultivation programs, where key morphobiological traits act as real-time markers for algorithms and help adjust irrigation and lighting regimes based on growth dynamics. This approach enables crop management in extreme environments, including long-duration space missions, while reducing monitoring costs and improving the accuracy of food resource planning.

## Figures and Tables

**Table 1:** Regression analysis equation models, where y is the mass of common millet (*Panicum miliaceum* L.) shoots (g) and x is the length of millet shoots (cm).

Graph	Model	Regression Model	Coefficient of Determination (R^2^)	Standard Error of Estimation
10	Linear	y = 0.012x − 0.015	0.749	0.011
10	Quadratic	y = (−0.021)x^2^ + 0.001x − 0.043	0.758	0.010
20	Linear	y = 0.019x − 0.077	0.806	0.051
20	Quadratic	y = (−0.004)x^2^ + 0.0005x + 0.034	0.825	0.048

**Table 2:** Regression analysis equation models for predicting the yield components of common millet (*Panicum miliaceum* L.).

Dependent Variable (y)	Model Predictors	Regression Model	Coefficient of Determination (R^2^)	Standard Error of Estimation
Weight of 1000 seeds, g	a—length of the main inflorescence, cm; b—number of all inflorescences on the plant, pcs; constant	y = 0.96a − 0.243b + 7.053	0.647	0.411
Weight of 1000 seeds, g	a—mass of grains in the main inflorescence, g; b—number of grains in the main inflorescence, pcs; c—length of the main inflorescence, cm; constant	y = 25.68a − 0.227b + 0.052c + 7.3	0.799	0.320
Number of productive inflorescences, pcs	a—number of all inflorescences on the plant, pcs; constant	y = 1.022a − 0.222	0.942	0.384
Total above-ground plant weight, g	a—number of all inflorescences on the plant, pcs; mass of the main inflorescence with grain, g; constant	y = 1.145a + 2.334b + 0.914	0.674	1.193
Total number of grains per plant, pcs	a—mass of grains in the main inflorescence, g; constant	y = 105.071a + 1.709	0.996	1.333
Weight of grains per plant, pcs	a—number of grains in the main inflorescence, pcs	y = 0.018a − 0.118	0.992	0.035

## Data Availability

The original contributions presented in this study are included in the article. Further inquiries can be directed to the corresponding author.

## Appendix A

**Table A1:** Morpho-biological features of common millet (*Panicum miliaceum*).

Features	Mean	Standard Deviation	Minimum	Maximum	Variation Coefficient, %
Shoot weight on day 10, g	0.06	0.02	0.02	0.12	34
Shoot weight on day 20, g	0.25	0.10	0.01	0.65	38
Shoot length on day 10, cm	6.3	1.5	3.5	11.3	23
Shoot length on day 20, cm	17.0	5.4	3	27.9	31
Number of days from sowing to the emergence of the first inflorescence	105.6	34.6	78	197	33
Plant height, cm	121.6	21	50	145	41
Number of internodes per shoot, pcs	8	1.1	6	10	14
Total above-ground plant biomass, g	6.2	2.6	0.5	12.8	42
Mass of shoots and leaves (excluding inflorescences), g	4.4	1.7	0.4	7.8	39
Length of the upper internode, cm	12.8	4.5	3.1	21.9	35
Length of the flag leaf, cm	34.4	9.9	17	43	29
Width of the flag leaf, cm	1.2	0.3	0.3	1.7	25
Length of trichomes on the upper part of the leaf sheath, mm	1.9	0.5	1.2	3.2	24
Length of trichomes at the base of the leaf, mm	2.2	0.4	1.6	3.1	18
Length of trichomes in the middle part of the leaf, mm	2.3	0.5	1.5	3.4	22
Length of the primary inflorescence, cm	22.9	4.8	13	29	18
Total number of inflorescences per plant, pcs	2.8	1.5	1	5	54
Number of productive inflorescences, pcs	2.6	1.5	1	5	58
Number of non-productive inflorescences, pcs	0.20	0.17	0	1	85

**Table A2:** Economical features of common millet (*Panicum miliaceum*).

Features	Mean	Standard Deviation	Minimum	Maximum	Variation Coefficient, %
Mass of the primary panicle, g	0.85	0.47	0.10	1.73	55
Mass of productive lateral panicles, g	0.93	0.72	0	3.40	78
Mass of non-productive lateral panicles, g	0.06	0.06	0	0.39	94
Total grain mass per plant, g	0.34	0.29	0	1.37	86
Grain mass in the main panicle, g	0.22	0.18	0	0.74	83
Grain mass from lateral panicles, g	0.23	0.18	0	0.76	78
1000-seed weight, g	8.61	0.65	7.49	9.56	8
Harvest index	0.06	0.01	0.01	0.22	23
Yield, kg/m^2^	0.31	0.19	0.08	0.78	63

**Table A3:** Technological features of common millet (*Panicum miliaceum*).

Features	Mean	Standard Deviation	Minimum	Maximum	Variation Coefficient, %
Average grain weight from the lower part of the panicle, g	0.009	0.002	0.005	0.011	18
Average grain weight from the middle part of the panicle, g	0.009	0.001	0.005	0.011	16
Average grain weight from the upper part of the panicle, g	0.009	0.002	0.003	0.010	18
Number of grains in the main panicle, pcs	24.4	17.4	0	80	71
Number of grains in lateral panicles, pcs	23.7	19.4	0	77	82
Length of grains from the lower part of the panicle, mm	3.2	0.2	3.0	3.6	5
Length of grains from the middle part of the panicle, mm	3.2	0.2	2.8	3.5	6
Length of grains from the upper part of the panicle, mm	3.2	0.2	2.8	3.5	6
Width of grains from the lower part of the panicle, mm	2.7	0.2	2.4	3.0	6
Width of grains from the middle part of the panicle, mm	2.6	0.2	2.3	2.9	6
Width of grains from the upper part of the panicle, mm	2.6	0.2	2.2	2.9	7
Grain length-to-width ratio index in the lower part of the panicle, mm	0.83	0.06	0.75	0.95	7
Grain length-to-width ratio index in the middle part of the panicle, mm	0.83	0.06	0.73	0.95	7
Grain length-to-width ratio index in the upper part of the panicle, mm	0.83	0.05	0.71	0.9	6
